# Diagnosis of Diseases of the Brain and of the Spinal Cord

**Published:** 1886-07

**Authors:** 


DIAGNOSIS OF DISEASES OF THE BRAIN AND OF THE SPINAL CORD. By W. R. Gowers, M. D., F. R. C. P., Assistant Professor of Clinical Medicine in University College, Physician to University College Hospital and to the National Hospital for the Paralyzed and Epileptic. New York: William Wood & Company. 1885.

Those who would understand what is known of the nervous system must first learn something of the structure and functions of those great nerve centres, the brain and spinal cord, with their departures from their normal physiological conditions. No proper comprehension of neurology is attainable without likewise investigating the ganglionic developments of the great sympathetic system, but as this is not included in the present undertaking, we may leave this halo of light, which is surrounded by another great circle of obscurity in nervous phenomena, and direct our attention more immediately to the cerebro-spinal system.

In view of the importance attached to the elementary principles involved in the study of cerebration and spinal phenomena, the work of Gowers, in diagnosticating some of their fundamental disorders, supplies much valuable information, not only for the student of neurology, but for the general practitioner of the healing art.

If the ignorance on the part of a large proportion of the medical profession as to the details of the anatomy and physiology of the brain and spinal cord had been more fully recognized in presenting his delineations of their disorders, this book would have proved of much greater utility, since many points are taken for granted which require elucidation for those of average attainments in this department of physiology.

The diagrams of the brain and the different sections of the spinal cord with the description of their functions afford a good basis for the comprehension of most of the phenomena presented, but it must strike the critical reader that there is some lack of precision in differentiating the several disorders, so as to leave a doubt of the exact relations of certain effects with their causes. Recent observations upon the local cerebral origin of remote nerve manifestations do not appear to have received due consideration in the course of the author's remarks upon the dependencies of brain lesions and other abnormal structure developments.

For instance, at the top of the 80th page we find that "paralysis of the face is the result of disease of the fibres or nucleus of the facial nerve or of the motor path between the facial nucleus and the cortex. This, as we have seen, lies to the inner side of the limb-path in the crus, and in front of it in the internal capsule, occupying the angle at the junction of the anterior and posterior parts of the capsule. The loss of power is on the same side as a lesion of the nerve fibres or of the nucleus in the pons, but, since the upward path decussates just above the nucleus, a lesion of the upper part of the pons, of the crus or of the hemisphere, causes paralysis of the face on the side opposite to the lesion."

It will be seen that, while this description of paralysis presents clearly the history of the associated lesions, there is not a distinct appreciation of the etiological factor as referable to the nerve centre. The same want of differentiation might be pointed out in various other parts of the work, and it is exactly in this phase of nervous disability that precision is requisite for the proper comprehension of such impairment or obliteration of the nerve-functions. In other words, it is not generalization but specialization that is called for by the student of neurology at the present day.

It is patent to all who have scrutinized closely the progress of medical philosophy during the past decade, that the greatest drawbacks to the elucidation of the etiology of diseases grow out of deficient acquaintance with the role of nerve-force and its modifications in originating the train of disorders which are concerned in the incipiency of structural derangements, constituting the different departures from health, known as diseases of various forms. The knowledge of gross changes in the tissues has gone ahead of the special histological modifications of structure; and while we have a fair acquaintance with pathological anatomy, comparatively small advances have been made in pathological physiology. The perversion of function from the action of the causative element of disease upon minute ramifications of the fibrillæ of nerves is doubtless the initial step in the train of disorder, which leads ultimately to the different phases of development according to the organ involved or the nature of the elements which enter into its minute histological structure. It must be apparent to all who have kept up with neurological investigations that we need more systematic and definite data connected with the origin, progress and results of so styled nervous disorders in like manner as of other types of diseases.

